# Clays as dietary supplements for swine: A review

**DOI:** 10.1186/s40104-015-0037-9

**Published:** 2015-08-22

**Authors:** Mohana Devi Subramaniam, In Ho Kim

**Affiliations:** Department of Animal Resource and Science, Dankook University, No. 29 Anseodong, Cheonan, Chungnam 330-714 South Korea

**Keywords:** Animal production, Clay, Clinoptilolite, Feed additive, Montmorillonite, Zeolite

## Abstract

Clays are crystalline, hydrated aluminosilicate molecules composed of alkali and alkaline earth cations along with small amounts of various other elements. The best-known are montmorillonite, smectite, illite, kaolinite, biotite and clinoptilolite. The molecules in these clays are arranged in three-dimensional structures creating internal voids and channels capable of trapping a wide variety of molecules. As a result of this structure, clay minerals are regarded as a simple and effective tool for the prevention of the negative effects of many toxic compounds. Dietary supplementation with clays has been shown to improve weight gain and feed conversion in pigs. Where improvements in performance have been noted, one of the most likely explanations for the improvement is the fact clays increase nutrient digestibility. Clays reduce the speed of passage of feed along the digestive tract which allows more time for digestion. Feeding clays also causes morphological changes in the intestinal mucosa such as an increase in villus height and an increase in the villus height to crypt depth ratio. These changes increase the surface area of the gastrointestinal tract thus increasing nutrient digestibility. Several studies have indicated that feeding clay reduces the incidence, severity and duration of diarrhea in pigs. The mechanism for the reduction in diarrhea is likely due to increases in the numbers of Bifidobacteria and Lactobacillus and decreases in Clostridia and E. coli in the small intestine of pigs fed clays. In addition, the numbers of pigs born alive and weaned, birth weight and weaning weight have been shown to be higher for sows fed clays. Several studies have indicated that clays can help mitigate the effects of mycotoxins. The aim of the present review is to focus on the various clays which have been given attention in recent research and to discuss their potential to improve pig performance.

## Introduction

Antibiotics are compounds used as growth promoters in animal feeds to decrease the animal’s susceptibility to infectious agents [1]. The use of antibiotics has been forbidden in the European Union since 2006 and many other countries are considering similar bans. As a result, intensive research has been focused on the development of alternative feeding strategies that can maintain pig performance and health [2–4]. One product class that has been suggested as an alternative feed additive for use in the pig industry is the inert clays [5–9].

Clays are crystalline, hydrated aluminosilicate molecules composed of alkali and alkaline earth cations along with small amounts of various other elements [10]. The molecules in these clays are arranged in three-dimensional structures creating internal voids and channels capable of trapping a wide variety of molecules [9]. As a result of this structure, clay minerals are regarded as a simple and effective tool for the prevention of the negative effects of toxic compounds.

Clays added to the diet can bind and immobilize toxic materials in the gastrointestinal tract of animals and reduce their toxicity. Several studies have confirmed the decontamination properties of clays against mycotoxins [11–17], heavy metals [18–24], and other toxins [25]. The adsorption capacity for any specific clay is determined by the fine structure of the clay particles, their surface properties and exchangeable ions [4]. Clays have also been shown to have a significant influence on growth, nutrient digestibility and the reproductive performance of swine. The aim of the present review is to focus on the various clays which have been given attention in recent research and to discuss their potential to improve pig performance.

### Structure of clays

Clays are naturally occurring minerals with a particle size less than 2 um in diameter [10]. They are composed mainly of phyllosilicates [10]. Phyllosilicates occur in layers and are classified according to the type of layers they possess, their interlayer content, the electronic charge of the layers and their chemical formula [4]. The structure of a phyllosilicate is based on a tetrahedral sheet of cations (commonly Si^4+^, Al^3+^ and Fe^3+^) and octahedral sheets of cations (commonly Al^3+^, Fe^3+^, Mg^2+^ and Fe^2+^). These layers have been classified into 1:1, 2:1 or framework structures [10].

Clays with a 1:1 layer structure typically have a tetrahedral Si sheet covalently bound to an octahedral Al sheet. Examples of this type of clay are kaolinite, dickite and nacrite, which belong to the kaolin group [10]. Clays classified as 2:1 layer structures have an octahedral sheet (Al, Mg or Al and Mg) between 2 tetrahedral Si sheets. Examples of this type of clay are montmorillonite, saponite, hectorite and beidellite which belong to the smectite group as well as illite, chlorite and vermiculite [10]. Zeolite is an example of a clay with a framework structure. These are a three dimensional tetrahedral structure of SiO_4_^4−^_,_ and AlO_4_^5−^ linked through shared oxygen atoms [10].

The structure and composition of the clay determines their physical and chemical properties. For example, smectites and illite have spaces between their layers that can expand to accommodate water and cations, allowing exceptional water absorption. As a result, they are able to absorb up to half their mass in water [10]. Monmorillonite is formed by a 2:1 condensation of layers of octahedrally coordinated aluminum sandwiched between two layers of tetrahedrally coordinated silica [26]. Montmorilonite has a electronic charge imbalance due to the replacement of Al^3+^ for Si^4+^ in the tetrahedral layer and Mg^2+^ or Zn^2+^ for Al^3+^ in the octahedral layer resulting in a negative charge at the clay surface. Monmorillonite can adsorb organic materials either on its surface or within its inter-laminar spaces through an exchange of cations present in these spaces, Due to these physical-chemical properties, montmorillonite has been extensively used in animal diets to promote production, reduce the detrimental effects induced by mycotoxin-contaminated feeds and protect against enteric diseases in swine [26].

Smetities have medium to high cation exchange capacity, high surface area, high adsorption capacity and high viscosity. Kaolin minerals (kaolinite, dickite, nacrite and halloysite) have minimal net charge but have properties such as very low cation exchange capacity, low surface area, and low adsorption capacity. Zeolites have three dimensional channel surfaces that can trap molecules according to the dimensions of the channel. Exchange of cations and water takes place within the three-dimensional channel structures present in the zeolite. They have a negative charge, high exchange capacity for certain cations (i.e. NH_4_^+^) with their selectivity a function of their pore size and capacity to adsorb contaminants.

### Use of clays to improve pig growth performance

The strategy of using clays to improve pig performance has been given considerable attention in research. Many studies have documented a significant improvement in weight gain and feed conversion in pigs fed diets supplemented with clays [6, 7, 26–31]. Improvements have been noted for both weanling pigs and growing-finishing pigs.

Yan et al. [28], reported that supplementation of the diet with 3 g/kg of a clay composite comprised of 72.6 % SiO_2_, 8.18 % Al_2_O_3_, 9.42 % Fe_2_O_3_, 5.25 % K_2_O, and 1.41 % Na_2_O could increase weanling pig performance (Table 1). In their study, weight gain was increased by 8.5 % while feed conversion was improved by 5.4 %. Trckova et al. [6] reported a 45 % increase in weight gain and a 16.9 % improvement in feed conversion ratio in weaned pigs fed diets supplemented with 1 % kaolin. Duan et al. [26] fed weaned pigs graded levels of montmorillonite (0 to 5 %) and reported that weight gain was unaffected by dietary treatment while feed intake linearly declined with increasing dietary montmorillonite which resulted in a significant improvement in feed conversion as the level of montmorillonite increased.

**Table 1:** Effect of clay supplementation on the performance of weanling pigs

Items	Control	Tylosin	Clay (3 g/kg)^1^	Clay (6 g/kg)^1^	SEM
Day 0 to 7					
Weight gain, g/day	258	295	288	257	15
Feed intake, g/day	391	395	399	405	24
Feed efficiency	0.66^b^	0.75^a^	0.72^a^	0.64^b^	0.02
Day 7 to 21					
Weight gain, g/day	429	458	466	432	20
Feed intake, g/day	554	546	591	560	42
Feed efficiency	0.78	0.84	0.80	0.77	0.05
Day 21 to 35					
Weight gain, g/day	526	580	567	517	28
Feed intake, g/day	935	913	944	916	37
Feed efficiency	0.56^b^	0.64^a^	0.60^a,b^	0.56^b^	0.03
Day 0 to 35					
Weight gain, g/day	434	474	471	431	24
Feed intake, g/day	674	663	694	672	35
Feed efficiency	0.64^b^	0.72^a^	0.68^a,b^	0.64^b^	0.03

Yan et al. [28]

^a,b^Values significant at *P*<0.05 level

^1^Clay comprised of 72.6 % SiO_2,_ 8.18 % AL_2_O_3_, 9.42 % Fe_2_O_3_, 5.25 % K_2_O and minor amounts of other minerals

Papaioannou et al. [29] conducted an experiment in which growing pigs were fed an unsupplemented control diet or similar diets supplemented with antibiotics (50 ppm enrofloxacin for weaners, 60 ppm salinomycin for grower-finishers) 2 % clinoptilolite or the two additives in combination at the same levels as those fed separately (Table 2). The diets were fed from weaning at 25 days of age until the pigs reach market weight at 161 days of age. From day 25 to 70, weight gain and feed conversion were significantly improved for pigs fed clinoptilolite. However the magnitude of the response was less than that obtained for pigs fed the antibiotics. In addition, it was evident that younger pigs responded more to clinopitolite than older pigs.

**Table 2:** Effect of clinoptilolite on the performance of growing and finishing pigs

Items	Treatment					*P* values		
	Control	Antibiotic^c^	Clinoptilolite^d^	Combination	SD	Clinoptilolite	Antibiotic	C x A
Day 25 to 70								
Weight gain, kg/d	0.34^c^	0.40^ab^	0.39^b^	0.41^a^	0.032	<0.01	<0.01	0.14
Feed intake, kg/d	0.68^a^	0.64^b^	0.68^a^	0.66^ab^	0.024	0.35	0.02	0.34
Feed conversion	2.02^a^	1.59^c^	1.75^b^	1.60^c^	0.19	<0.01	<0.01	<0.01
Day 71 to 112								
Weight gain, kg/d	0.63^c^	0.69^ab^	0.66^bc^	0.71^a^	0.04	0.09	<0.01	0.72
Feed intake, kg/d	1.79	1.76	1.77	1.69	0.11	0.33	0.22	0.60
Feed conversion	2.84^a^	2.55^bc^	2.69^ab^	2.38^c^	0.22	0.03	<0.01	0.92
Day 113-161								
Weight gain, kg/d	0.83^b^	0.90^a^	0.85^b^	0.91^a^	0.04	0.29	<0.01	0.21
Feed intake, kg/d	2.69^a^	2.56^b^	2.64^ab^	2.61^ab^	0.10	0.89	0.15	0.24
Feed conversion	3.25^a^	2.86^b^	3.12^a^	2.87^b^	0.19	0.24	<0.01	0.16
Day 25 to 161								
Weight gain, kg/d	0.60^c^	0.67^a^	0.64^b^	0.69^a^	0.03	0.04	<0.01	0.21
Feed intake, kg/d	1.74	1.67	1.72	1.67	0.06	0.56	0.12	0.58
Feed conversion	2.88	2.51	2.70	2.45	0.19	0.04	<0.01	0.12

Papaioannou et al. [29]

^a,b^Values significant at *P*<0.05 level

^c^Antibiotic was a combination of enrofloxacin and salinomycin

^d^Clinopilolite fed at 2 % of the diet

Prvulovic et al. [7] reported a 7 % increase in weight gain and a 1.4 % improvement in FCR in growing pigs fed diets supplemented with 0.5 % clinoptilolite during the growing period while no improvement was noted during the finishing period. Yu et al. [20] reported that weight gain, feed intake and feed conversion ratio were improved by 8.9, 3.9 and 4.8 % as a result of feeding 0.5 % montmorillonite to growing pigs. Li and Kim [27] reported that growing pigs fed a diet supplemented with 0.5 % sericite had 6.6 % higher weight gain and a 5.1 % better feed conversion ratio compared with pigs fed an unsupplemented diet.

Alexopoulos et al. [30] compared the performance of pigs fed 2 % clinopilolite with the performance of pigs fed an unsupplemented control from 25 days of age until market weight at 161 days of age. They reported a 5.3 % improvement in weight gain for pigs fed clinopilolite. Again, the response was greater in younger pigs than older pigs. Pond et al. [31] reported that weight gain was increased by 14.3 % and feed conversion by 2.9 % as a result of feeding 2 % clinoptilolite to growing pigs.

Unfortunately, the effects of clay supplementation on pig performance have been inconsistent and there are many studies which have noted no improvements in pig performance as a result of feeding clays. Song et al. [5] reported no improvement in weight gain or feed conversion as a result of feeding 0.3 or 0.6 % smectite to weaned pigs. In a subsequent experiment, they compared smectite, kaolinite and zeolite fed individually and in all possible combinations to total 0.3 % of the diet and observed no improvements in pig performance. Xia et al. [32] reported no effect on pig performance from including 0.15 % montmorillonite in the diet of weanling pigs.

Chen et al. [8, 9] observed no improvement in growing-finishing pig performance for pigs fed 0.5 % Biotite (61.9 % SiO_2_, 23.19 % Al_2_O_3_, 3.97 % Fe_2_O_3_ and 3.35 % Na_2_O). Similarly, Thacker [33] fed 0, 0.25, 0.5 or 0.75 % Biotite during the growing period and 0, 0.5, 1.0 or 1.5 % during the finishing period and observed no improvement in pig performance. Yan et al. [34], fed the same composite clay as Yan et al. [28] and found no improvement in growing-finishing pig performance. In addition, Lin et al. [18], Yu et al. [20], Poulsen and Oksbjerg [35], Shurson et al. [36], Parisini et al. [37], Abranches et al. [38] and Han and Thacker [39] reported no improvements in pig performance as a result of dietary inclusion of clays.

As noted previously, the effect of clays on pig performance is inconsistent. In general, younger pigs respond more to dietary clay supplementation than older pigs [6, 28–30]. In addition, the level of supplementation affects the response. Supplementation of diets with 1 to 3 % clay is recommended [4, 6].

### Use of clays to improve nutrient digestibility

Where improvements in pig performance have been noted, one of the most likely explanations for the improvement in performance is the fact that dietary clay supplementation has been shown to increase nutrient digestibility [8, 9, 27, 28, 34–37]. Li and Kim [27] reported that dietary supplementation with 0.5 and 1.0 % sericite increased the total tract digestibility of dry matter by 3.9 and 7.5 %, as well as the digestibility of nitrogen by 4.9 and 5.7 % for growing pigs (Table 3). The digestibility of calcium and phosphorus were increased by 5.0 to 19.1 % when pigs were fed diets containing 0.5 or 1.0 % sericite. The total tract digestibility of energy was unaffected by treatment.

**Table 3:** Effects of including sericite in diets fed to growing pigs on the apparent total tract digestibility of nutrients

Items	Level of sericite, %				
	0.0	0.5	1.0	SE	*P* value
Dry matter	0.76	0.80	0.82	0.009	0.01
Nitrogen	0.79	0.83	0.83	0.008	0.03
Energy	0.79	0.78	0.82	0.013	0.19
Calcium	0.56	0.59	0.60	0.007	0.02
Phosphorus	0.42	0.50	0.50	0.005	0.03

Li and Kim [27]

Yan et al. [34] reported that the digestibility of dry matter, nitrogen and energy was quadratically increased when growing-finishing pigs were fed 0, 3 or 6 g/kg of a clay composite (Table 4). In a subsequent experiment, Yan et al. [28] reported significant improvements in dry matter and nitrogen digestibility for weaned pigs following 7 days of feeding the clay composite while no difference was observed after 21 days of treatment. Parisini et al. [37] reported that the inclusion of 2 % sepiolite improved protein and energy digestibility of weaned pigs by 6.1 and 5.3 % compared with control pigs.

**Table 4:** Effects of clay supplementation on nutrient digestibility in growing-finishing pigs^1^

Items	Level of clay (g/kg)				*P* - value	
	0	3	6	SE^2^	Linear	Quadratic
Dry matter	0.77^b^	0.82 ^a^	0.77 ^b^	0.009	0.91	<0.01
Nitrogen	0.76 ^b^	0.79 ^a^	0.74 ^b^	0.006	0.05	<0.01
Energy	0.81^ab^	0.83 ^a^	0.78 ^b^	0.013	0.13	0.04

Yan et al. [34]

^a,b^Values significant at *P*<0.05 level

^1^Clay comprised of 72.6 % SiO_2,_ 8.18 % AL_2_O_3_, 9.42 % Fe_2_O_3_, 5.25 % K_2_O and minor amounts of other minerals

Chen et al. [8] reported that nitrogen and calcium digestibilities were increased by the addition of 0.5 % Biotite V to the diet while dry matter and phosphorus digestibilities were unaffected. In a subsequent experiment, Chen et al. [9] reported significant improvements in the digestibility of dry matter, nitrogen, calcium and phosphorus as a result of supplementation with either 1 or 2 % Biotite V.

In contrast, Thacker [33] and Han and Thacker [39] reported no improvement in nutrient digestibility as a result of including clays in the diet of growing-finishing pigs. Shurson et al. [36] reported negative effects on nutrient digestibility when diets containing 0, 2.5, 5.0 or 7.5 % clinopilolite were fed to starter pigs. Poulsen and Oksbjerg [35] also reported negative effects on dry matter and nitrogen digestibility as a result of feeding 3 % clinoptilolite to young growing pigs while Fokas et al. [22] indicated that clay (organozeolites) had no influence on nutrient utilization in growing pigs fed a zearalenol contaminated feed.

Where improvements in nutrient digestibility have been observed, several mechanisms have been proposed to explain the effect. Firstly, it has been proposed that clays reduce the speed of passage of feed along the digestive tract [4, 6, 29] which would allow more time for digestion. Secondly, some authors have put the improvement in protein and energy retention as a result of clay supplementation down to increased activity of pancreatic enzymes [6]. The increased activity is proposed to result from the fact that pancreatic enzymes bind to the surface of the adsorbents and form complexes that are active within a wider range of pH in the digestive tract [40]. Finally, feeding clay minerals can cause morphological changes in the intestinal mucosa [32, 41]. Xia et al. [32, 41] reported that the villus height and the villus height to crypt depth ratio were 19.1 and 37.1 % higher in pigs fed diets supplemented with 0.2 % montmorillonite compared with a control (Table 5). An increase in villus height increases the surface area for nutrient absorption thus increasing nutrient digestibility.

**Table 5:** Effect of montmorillonite on the intestinal morphology in the jejunum

Items	Control	Montmorillonite
Villus height (um)	440^a^	524^b^
Crypt depth (um)	356	309
Villus height: crypt depth	1.24^a^	1.70^b^

Xia et al. [32]

^a^ and ^b^ Values significant at *P*<0.05 level

### Use of clays to reduce the incidence of diarrhea in swine

Where improvements in pig performance have been noted, another potential explanation for the improved performance of pigs fed diets containing clays is that the health status of pigs can be improved by the adsorption ability of clay. Several studies have indicated that feeding clays to pigs reduces the incidence, severity and duration of diarrhea [5, 6, 29, 41]. Song et al. [5] reported that the diarrhea score was improved 16.5 and 21.8 % by feeding 0.3 and 0.6 % smectite (Table 6). In the same study, the frequency of diarrhea was reduced 70.8 and 75.0 % by feeding 0.3 or 0.6 % smectite. Xia et al. [41] reported that the incidence of diarrhea was reduced from 19.1 to 5.4 % as a result of feeding 0.2 % montmorillonite to weaned pigs. Papaioannou et al. [29] reduced the diarrhea scores of weaner pigs from 0.93 to 0.66 by feeding 2 % clinoptilolite. In contrast, Abranches et al. [38] reported no improvement in diarrhea scores from feeding 0.3 % smeectite to weaner pigs.

**Table 6:** Effect of smectite on the diarrhea scores of pigs experimentally infected with a pathogenic *E. coli*

Items	Level of smectite, %			*P* value
	0	0.3	0.6	
Diarrhea score^1^	1.99	1.66	1.62	<0.05
Diarrhea days^2^	17	5	4	<0.05
Frequency^3^, %	24	7	6	<0.05

Song et al. [5]

^1^diarrhea score = 1, normal feces, 2, moist feces, 3, mild diarrhea, 4, severe diarrhea, 5, watery diarrehea

^2^diarrrhea days = number of pig days with diarrhea score greater than 3

^3^Frequency = diarrhea x 100/pig days

There are several potential mechanisms through which clays may reduce the incidence of diarrhea in swine. Clays such as smectites and illite have spaces between their layers that can expand to accommodate water and cations, allowing exceptional water absorption. As a result, they are able to absorb up to half their mass in water [10]. This would result in the presentation of a firmer feces and thus influence the presentation of diarrhea directly.

Clays have also been shown to alter the microbial population in the gastrointestinal tract resulting in a more favorable gut microflora. Xia et al. [41] reported that supplementation with 0.2 % montmorillonite significantly reduced the viable counts of E.coli in the jejunum of weanling pigs. In a subsequent experiment, Xia et al. [32] reported increases in the numbers of Bifidobacteria and Lactobacillus and decreases in Clostridium and E. coli in the small intestine of pigs fed 1.5 g/kg montmorillonite (Table 7). Li and Kim [27] reported that supplementation of 0.5 and 1.0 % sericite decreased the fecal E .coli population by 12.6 and 11.6 % and decreased fecal Lactobacillus counts by 9.5 and 14.7 % respectively while Wang et al. [42] reported that fecal Lactobacillus counts were increased and E. coli counts were reduced in weaned pigs fed diets supplemented with montmorillonite clay. In contrast, Thacker [33] reported no difference in Lactobacillus, Enterobacteria or Salmonella counts as a result of feeding graded levels of montmorillonite. Where alterations in the makeup of the gut microflora occur, it has been suggested that the ion exchange capacity of clays may modify the characteristics of the intestinal environment such as pH or oxidation state, thus influencing the growth of specific bacteria [5].

**Table 7:** Effect of montmorillonite on the intestinal flora of weanling pigs (log_10_ CFU/g)

Items	Control	Montmorillonite
Total anerobes	8.4	8.2
Total anaerobes	9.4	9.3
Bifidobacteria	6.9	7.4
Lactobacillus	7.9	8.2
Clostridium	6.5	5.8
*E. coli*	7.8	7.4

Xia et al. [32]

Additional mechanisms through which clays may reduce the incidence of diarrhea were reviewed by Song et al. [5]. They suggested that clays may attract bacterial cells with enough physical force to tear the cell membrane resulting in lysis of the bacterial cells. Clays can also adsorb or detoxify bacterial toxins and thus protect pigs from increases in intestinal permeability and damage by toxins. In addition, clays may adhere to the gastrointestinal mucous membranes and reinforce the physical mucous barrier resulting in some protection against enteric diseases caused by bacteria.

Another mechanism through which clays can reduce the incidence of diarrhea is through increasing the numbers of goblet cells. Goblet cells produce mucins in the small intestine and these proteins comprise the bulk of the mucus layer which acts as the first line of defense against eneteric infections. Abranches et al. [38] observed a tendency for an increase in goblet cell size and number in the ileum of pigs fed smectite. The modest increase in goblet cell size and number when clays were fed may at least partially explain the reduction in diarrhea observed in pigs [38].

Concerning newborn animals, the administration of zeolites appears to reduce the incidence of diarrhea through the enhancement of passive immunity, as they increase the net absorption of colostrum immunoglobulins in pigs [43].

### Other mechanisms to explain the growth promoting effects of clays

It has been suggested that growth and feed efficiency were improved due to the reduced microbial production of ammonium in the intestines when clays are fed [36]. This lowers the nutritional demands for cell renewal and thereby increases the amount of nutrients available for growth [35].

Another potential explanation for the improved performance of pigs fed clays is the result of increased growth hormone levels. Yu et al. [21] reported that serum growth hormone peak, base-line levels and mean levels were increased by 117.1, 42.8 and 51.7 % in pigs fed 0.5 % montmorillonite.

### Effect of clays on carcass traits

Little research has been conducted to determine the effects of dietary inclusion of clays on swine carcass traits. Thacker [33] reported that feeding clays did not affect dressing percentage, lean yield, loin fat, loin lean or carcass value index of pigs fed graded levels of Biotite. Similarly, Han and Thacker [39] reported no effects from feeding Hwangto clay on the percentages of tenderloin, bacon, fat and bone, skirt, fresh ham or ribs. However, pigs fed Hwango had a higher percentage of loin. Yan et al. [34] reported no effects on meat quality except that meat firmness was increased by 20.2 % for pigs fed 6 g/kg composite clay (Anion).

### Reproduce performance of pigs fed clays

Considerably less research has been conducted to determine the effects of clays on the reproductive performance of pigs than has been conducted to determine the effects of clays on growth performance. Papaioannou et al. [44] reported that the farrowing rate and percentage of sows returning to estrus was 78.7 and 14.1 % in control sows and 86.2 and 10.3 % in sows fed 2 % zeolite (Table 8). In addition, the percentage of sows with inappetence, pyrexia, and mastitis was lower in sows fed zeolite than control sows. However, the percentage of sows with vaginal discharges was lower in control sows than in sows fed zeolite.

**Table 8:** Effect of zeolites on sow fertility and diseases

Items	Control	Zeolite
Return to estrus	14.1	10.3
Farrowing rate	78.7	86.2
Inappetence	40.8	35.1
Pyrexia	29.1	26.1
Mastitis	17.5	14.4
Vaginal discharge	6.8	8.1

Papaioannou et al. [44]

Kyriakis et al. [45] reported that the number of pigs born alive and weaned were 9.6 and 8.72 in control sows compared with 10.32 and 9.49 in sows fed 2 % zeolite (Table 9). Preweaning mortality was 9.2 % for control sows and 8.0 % for sows fed zeolite. Birth weight and weaning weight were also significantly higher in piglets born to sows fed zeolite than control sows.

**Table 9:** The effects of clinoptilolite on the reproductive performance of sows

Items	Control	Clinoptilolite
Piglets born alive	9.60	10.32
Piglets weaned	8.72	9.49
Preweaning mortality, %	9.16	8.04
Birth weight, kg	1.34	1.44
Weaning weight, kg	6.04	6.28

Kyriakis et al. [45]

### Use of clay to reduce the effects of mycotoxins

One of the most important roles of clays for pigs is their ability to mitigate the effects of mycotoxins. Mycotoxins are toxic secondary metabolites of fungi commonly found on grains, which can cause severe negative impacts on swine health and performance. Clays may have the ability to diminish the impact of mycotoxins through their binding properties. Lindeman et al. [13] reported that inclusion of 0.5 % hydrated sodium calcium aluminosilicate or sodium bentonite in a diet with 840 ppm aflatoxin prevented most of the reductions in weight gain and feed intake observed in weanling pigs. Schell et al. [14, 15] reported similar findings.

Wang et al. [42], reported that the addition of montmorillonite clay to the diet can ameliorate the negative effects of dietary zearalanone and accelerate recovery of zearalanone toxicity in weaning pigs during and after zearalanone challenge (Table 10). In their experiment, nursery pigs were fed diets contaminated with 0, 0.2, 0.4 or 0.8 ppm zearalenone which depressed weight gain and negatively impacted feed efficiency. The addition of montmorillonite to the diet significantly improved the feed efficiency of pigs fed zeralanone. In addition, the total tract digestibility of nitrogen was improved by supplementation with montmorillonite while dry matter and energy digestibility were unaffected. Montmorillonite clay also improved the performance of growing gilts fed zearalenone contaminated diets. Wang et al. [42] reported significant improvements in weight gain and feed efficiency as a result of including 2 g/kg montmorillonite in diets contaminated with 0, 0.2, 0.4 or 0.8 ppm zearalenone (Table 11).

Zearalenone causes infertility, abortion and breeding problems in swine and can be found in cereal crops like maize and its byproducts. Zearalenone and its metabolites bind competitively to estrogen receptors which causes hypertrophy of the uterus [42]. Feeding clay resulted in a significant reduction in vulva length, width and area in gilts fed zearalenone (Table 11). Similar results were reported by Jiang et al. [46, 47].

**Table 10:** The effect of montmorillonite clay on the performance and total tract digestibility of nutrients in diets fed to nursery pigs after challenge with zearalenone (ZEA)^a^

ZEA, mg/kg	0		0.2		0.4		0.8		SEM	*P*- value				
Clay, g/kg	0	2	0	0.5	0	1	0	2		ZEA	MC	ZEA x MC	Lin	Quad
Performance														
Weight gain, g/day	643	628	622	635	610	634	581	608	26	0.03	0.29	0.21	0.03	0.21
Feed intake, g/day	821	839	920	896	884	929	870	825	20	0.01	0.17	0.14	0.18	0.04
Feed efficiency	0.79	0.75	0.68	0.71	0.69	0.68	0.67	0.74	0.017	0-.04	0.03	0.39	0.03	0.37
Apparent total tract digestibility coefficient, %														
Dry matter	0.848	0.839	0.820	0.848	0.803	0.836	0.798	0.835	0.01	<0.01	0.19	0.45	0.01	0.28
Nitrogen	0.827	0.833	0.797	0.833	0.804	0.842	0.786	0.826	0.02	<0.01	<0.01	0.39	0.04	0.34
Energy	0.856	0.854	0.859	0.859	0.818	0.875	0.829	0.878	0.01	0.45	0.87	0.27	0.38	0.22

^a^Wang et al. [42]

**Table 11:** The effects of montmorillonite clay on performance and reproductive tract characteristics of growing gilts after challenge with zearalenone (ZEA)

ZEA, mg/kg	0		0.2		0.4		0.8		SEM	*P*- value		
Clay, g/kg	0	2	0	2	0	2	0	2		ZEA	MC	ZEA x MC
Performance												
Weight gain, g/day	672	680	651	734	615	686	617	690	18	<0.01	<0.01	0.25
Feed intake, g/day	809	815	819	868	757	832	782	820	20	0.02	0.25	0.33
Feed efficiency	0.83	0.84	0.79	0.85	0.81	0.83	0.79	0.84	0.02	<0.01	<0.01	0.65
Vulva size												
Width, mm	19.18	19.17	21.09	19.72	22.26	20.10	23.39	20.09	0.6	<0.01	<0.01	0.22
Length, mm	22.70	22.35	25.92	23.14	26.25	23.12	27.52	23.96	0.8	0.01	<0.01	0.19
Area, mm^2^	217.52	214.01	273.27	229.17	292.16	232.26	321.88	242.37	12	0.02	0.01	0.34

Wang et al. [42]

### Environmental impact of feeding clays to pigs

Feeding clay to pigs may help to minimize the impact of swine production on the environment. Shurson et al. [36] and Poulsen and Oksbjerg [35] reported that the amount of nitrogen excreted in feces was significantly higher while urinary nitrogen excretion was lower in pigs fed zeolites. As urinary nitrogen is more volatile than fecal nitrogen, this change in excretion pattern would tend to reduce the amount of nitrogen lost to the environment from a swine operation.

**Fig. 1:**
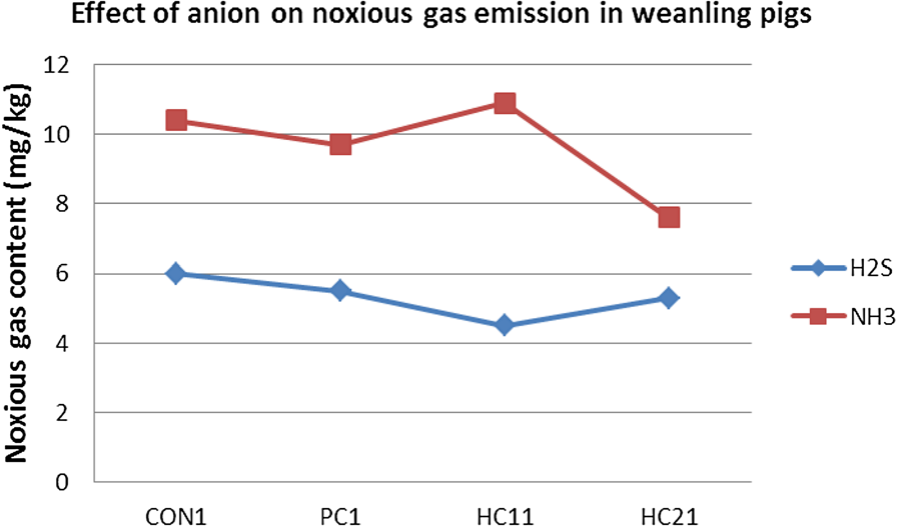
Effect of supplemental clay on noxious gas content in feces from weanling pigs. Dietary treatments were as follows: CON: basal diet, PC: basal diet with 44 mg/kg of Tylosin, HC1: basal diet with 3 g/kg Anion plus powder, HC2: basal diet with 6 g/kg Anion plus powder. In this study, the inclusion of HC2 treatment decreased NH3 emission (*P* < 0.05) compared with other treatments. No difference was observed on H2S. Nitrification of sludge is accelerated by the use of Anion, which selectively exchanges NH_4_^+^ from wastewater and provides an ideal growth medium for nitrifying bacteria that can oxidise NH_4_^+^ to nitrate. The supplementation of the diet with 3 g/kg Anion concomitantly decreased the noxious gas emission [28]

Yan et al. [28, 34] reported that hydrogen sulfide and ammonia levels were reduced in the feces of weanling pigs fed a composite clay (Fig. 1). They suggested that clay may selectively exchange ammonia from waste water and provide an ideal growth medium for nitrifying bacteria that can oxidise ammonia to nitrate.

Volatile fatty acids are responsible for a significant proportion of the odor arising from swine operations. Chen et al. [9] reported that fecal propionic acid, butyric acid and acetic acid were significantly reduced in pigs fed Biotite. They also reported reductions in fecal ammonia levels.

## Conclusions

Livestock production can make good use of resources, which contributes high quality nutrient to the human diet [48]. Dietary supplementation with clays has been shown to improve weight gain and feed conversion in pigs. Where improvements in performance have been noted, one of the most likely explanations for the improvement is the fact that dietary clay supplementation increases nutrient digestibility. Clays reduce the speed of passage of feed along the digestive tract which allows more time for digestion. Feeding clay minerals also causes morphological changes in the intestinal mucosa such as an increase in villus height and an increase in the villus height to crypt depth ratio. An increase in villus height increases the surface area for nutrient absorption thus increasing nutrient digestibility. Several studies have indicated that feeding clay reduces the incidence, severity and duration of diarrhea in pigs. The mechanism for the reduction in diarrhea is likely due to increases in the numbers of Bifidobacteria and Lactobacillus and decreases in the numbers of Clostridium and E. coli in the small intestine of pigs fed clay. In addition, the numbers of pigs born alive and weaned, birth weight and weaning weight have been shown to be higher for sows fed clay. Several studies have indicated that clays can mitigate the effects of mycotoxins. Clays can be used as an alternative to antibiotics for the prevention of diarrheal diseases and the enhancement of pig growth and reproduction during crucial periods.
